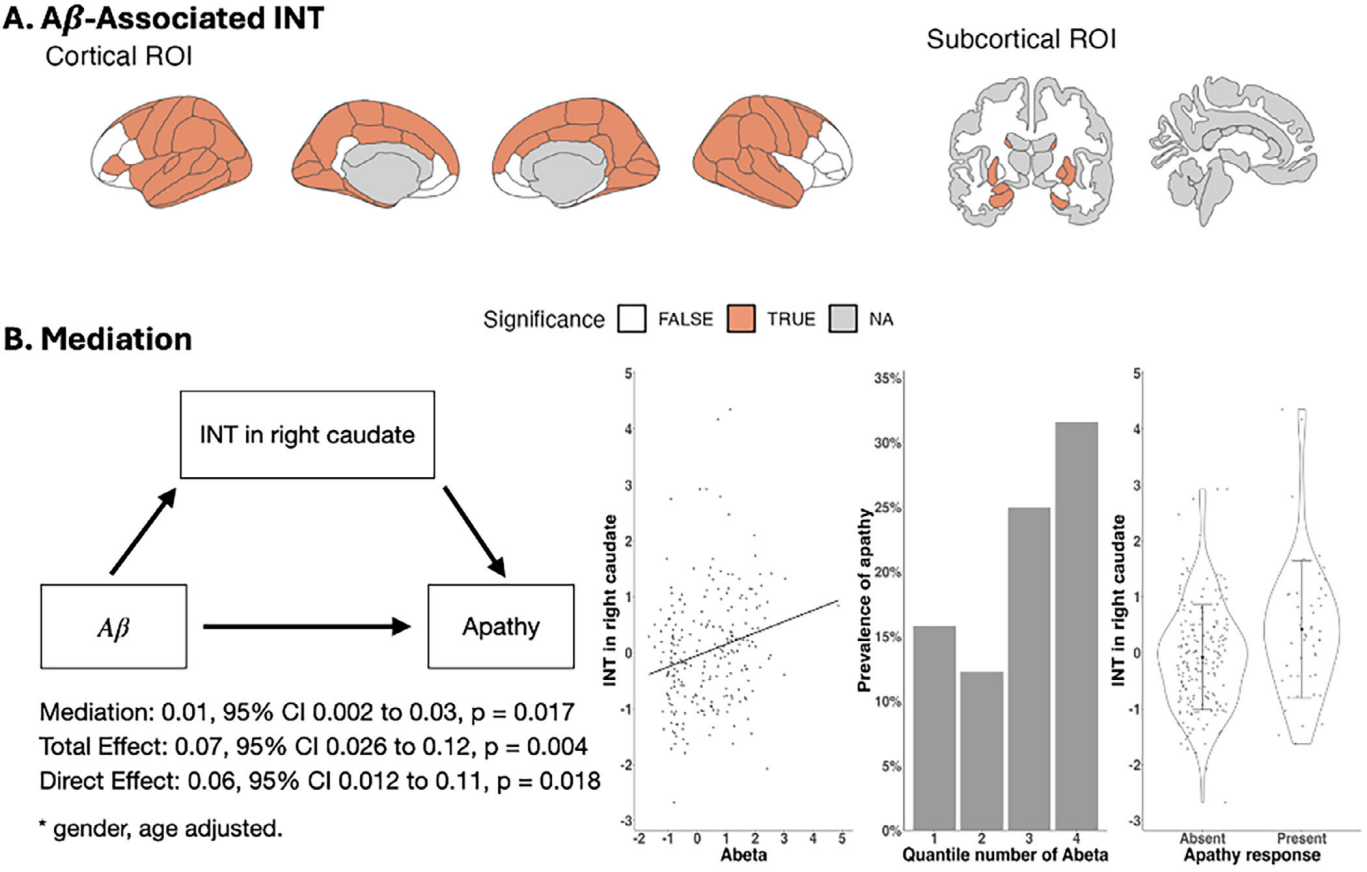# Intrinsic Neural Timescale Mediates Amyloid‐Related Apathy in Alzheimer’s Disease

**DOI:** 10.1002/alz70861_108820

**Published:** 2025-12-23

**Authors:** Yijin Wang, Hyun Kim, Aiying Zhang, Xi Zhu, Terry E. Goldberg, Seonjoo Lee

**Affiliations:** ^1^ Mailman School of Public Health, Columbia University, New York, NY USA; ^2^ Columbia University Irving Medical Center, New York, NY USA; ^3^ New York State Psychiatric Institute, New York, NY USA; ^4^ University of Virginia, Charlottesville, VA USA; ^5^ University of Texas at Arlington, Arlington, TX USA; ^6^ Division of Geriatric Psychiatry, New York State Psychiatric Institute, New York, NY USA

## Abstract

**Background:**

Apathy is one the most common symptoms in Alzheimer’s disease (AD). However, the neural mechanisms underlying apathy in AD remain poorly understood. Recent work has shown alterations in hierarchical order of the intrinsic neural timescale (INT), which reflects how persistently a brain region processes information over time, in AD and psychiatric disorders. However, the involvement of INT in AD‐related apathy has not been studied.

**Method:**

We analyzed 158 MCI and 69 AD participants from Alzheimer’s Disease Neuroimaging Initiatives (ADNI) with resting state fMRI, amyloid () PET and neuropsychiatric inventory data at baseline. The fMRI data were preprocessed using fMRIPrep (version v20.1.1). For each voxel, we calculated the autocorrelation function (ACF), and INT was then estimated as the area under the ACF during the initial positive period. ROI‐specific regional INT were calculated across 84 ROIs (68 cortical and 16 subcortical regions). Site‐effect was removed using ComBat. We performed regression analysis to test the effect of amyloid on the regional INT, and tested mediation of the regional INT on the effect of amyloid on apathy presence. All analyses were adjusted for age and sex, with false discovery rate correction applied for multiple comparisons.

**Result:**

We found INTs of 56 out of 84 ROIs were significantly associated with (Figure 1A). INTs of all cortical and subcortical ROIs increased as increased (except for that of the left frontal pole). INT of the right caudate significantly mediated in the effect on apathy (ACME=0.01, 95% CI: 0.002 to 0.03, *p* =0.017, Figure 1B); on average, for one‐standard‐deviation increase in, the probability of having apathetic symptoms, mediated by INT of right caudate, increases by 0.01. The findings remained similar after controlling for depression. Interestingly, we found a different mediation pathway for depression: right precuneus significantly mediated the effect of on depression (ACME=‐0.01, 95% CI: ‐0.023 to ‐0.001, *p* =0.033).

**Conclusion:**

This is the first demonstration of the mediating role of INT in AD‐associated apathy among elderly individuals with diverse clinical statuses of AD, providing potential insight into a neural substrate of apathy, independent of that of depression, in AD.